# Applying the Cognitive Walkthrough for Implementation Strategies Methodology to Inform the Redesign of a Selection-Quality Implementation Toolkit for Use in Schools

**DOI:** 10.21203/rs.3.rs-4505754/v1

**Published:** 2024-07-22

**Authors:** Kelsey Dickson, Olivia Michael, Amy Drahota, Aksheya Sridhar, Jessica Tschida, Jill Locke

**Affiliations:** San Diego State University; University of Washington; Michigan State University; Michigan State University; Michigan State University; University of Washington

**Keywords:** Implementation Strategy Design, Implementation Science: Community Participatory Approach, Human-Centered Design

## Abstract

**Background:**

Implementation strategies are key to enhancing translation of new innovations but there is a need to systematically design and tailor strategies to match the targeted implementation context and address determinants. There are increasing methods to inform the redesign and tailoring of implementation strategies to maximize their usability, feasibility, and appropriateness in new settings such as the Cognitive Walkthrough for Implementation Strategies (CWIS) approach. The aim of the current project is to apply the CWIS approach to inform the redesign of a multifaceted selection-quality implementation toolkit entitled ACT SMARTS for use in middle and high schools.

**Methods:**

We systematically applied CWIS as the second part of a community-partnered iterative redesign of ACT SMARTS for schools to evaluate the usability and inform further toolkit redesign areas. We conducted three CWIS user testing sessions with key end users of school district administrators, school principals, and educators.

**Results:**

Our CWIS application revealed that end users found ACT SMARTS acceptable and relevant but anticipate usability issues engaging in the ACT SMARTS process. Results informed the identification of eleven usability issues and corresponding redesign solutions to enhance the usability of ACT SMARTS for use in middle and high schools.

**Conclusions:**

Results indicated the utility of CWIS in assessing implementation strategy usability in service of informing strategy tailoring and redesign to improve alignment with user and setting needs. Recommendations regarding the use of this participatory approach are discussed.

## Background

Implementation strategies, or methods or techniques to enhance adoption, use, and sustainment of new innovations, are critical to facilitating implementation outcomes such as feasibility, acceptability, and appropriateness (1,2). There have been increasing efforts to develop and test implementation strategies, including discrete or singular strategies and multifaceted or two or more singular strategies (2). Yet, variable effects of implementation strategies, particularly multifaceted strategies, are common (3–6). The limited systematic or intentional design of implementation strategies considering the contexts in which they are deployed contributes to these limited effects, prompting increased calls and an emerging focus on more intentional, systematic development, and tailoring of implementation strategies (2). Fortunately, there have been increased efforts to develop feasible, pragmatic methods for designing and tailoring implementation strategies and a corresponding growing literature describing their application and impact (e.g., 6–8). The Cognitive Walkthrough for Implementation Strategies (CWIS) is one such method for assessing implementation strategy usability in service of informing tailoring and redesign of implementation strategies (10). Yet, additional use of this method is needed to inform further development and application, particularly in real-world practice. As such, the current work describes the application of the CWIS method as part of a broader project that aims to iteratively redesign a multifaceted selection-quality implementation toolkit for use in a novel context (i.e., middle and high schools).

### Cognitive Walkthrough for Implementation Strategies

CWIS is drawn from the human-centered design (HCD) field. HCD aims to develop or design products that are intuitive, effective, and match the needs and desires of its intended users and contexts (11). Towards that goal, HCD methods frequently involve evaluating and understanding user and context-specific needs and constraints, and iterative prototyping paired with ongoing user-testing to inform the development of appropriate, usable products (12,13). A primary goal and outcome of HCD is usability or the extent to which a product can be used by specified individuals to achieve specified goals in a specified context (14). While related to other implementation outcomes such as acceptability and feasibility relevant within implementation studies, usability is conceptualized as a determinant predictive of implementation outcomes (10).

Consistent with HCD, CWIS is designed to be a low cost, pragmatic method to evaluate implementation strategy usability, and has been applied to complex or multifaceted strategies. CWIS specifies six sequential tasks or steps. The first step of CWIS (**Step 1**) involves determining preconditions or the necessary situations or prerequisites required for an implementation strategy to be effective. Users then complete a hierarchical task analysis (**Step 2**) whereby tasks or steps comprised within the implementation strategy are articulated and specified. This includes identifying the behavioral or cognitive activities as well as how specifying the temporality and sequencing of tasks required to enact or carry out the implementation strategy. Next, task prioritization (**Step 3**) occurs whereby tasks that 1) are most important to be completed correctly or 2) where users are either most likely to encounter issues or commit errors, thereby impeding the execution or success of the implementation strategy, are identified and prioritized. Specifically, tasks identified in prior steps are rated on these two dimensions using a Likert scale ranging from “1” (unlikely to make an error/unimportant to complete correctly) to “5” (extremely likely to make an error/extremely important to complete correctly) by those with expertise or strong familiarity with the implementation strategy. Mean ratings are calculated to combine likelihood of issue/error and completion importance. The highest rated tasks are then selected and prioritized. While there are no task selection cutoffs articulated for the CWIS method, raters make the final task selections based on those identified as highest importance.

The fourth step (**Step 4**) involves converting the prioritized tasks into a series of scenarios and subtasks which are used for pragmatic user testing (**Step 5**) with a sample of representative end users. During testing sessions, user testing participants are presented with the developed scenarios and tasks, asked to cognitively enact and reflect on presented tasks and then complete quantitative ratings of their anticipated likelihood of success supplemented and provide corresponding qualitative justifications for ratings. Following user testing, the last step (**Step 6**) involves usability issue identification, prioritization, and classification, which points to key areas for implementation strategy redesign. Since its inception, there are increasing incorporation of CWIS into new proposals and projects (e.g., 15–17) but published work detailing its application remain limited, particularly independent applications outside of method developers. Further application and examples of application of CWIS are needed to inform and develop this novel method.

### Current Aims

The current work provides a case study of the application of CWIS to identify key usability issues to inform further redesign of a multiphase and multi-activity selection-quality implementation toolkit entitled Adoption of Curricular supports Toolkit: Systematic Measurement of Appropriateness and Readiness for Translation in Schools (ACT SMARTS; described below) for use in middle and high schools within the context of autism-EBP implementation. Specific guiding research questions as part of the CWIS application include:

1. To what extent does the CWIS methodology highlight end users’ understanding of the ACT SMARTS phases and activities presented to them?
2. To what extent does CWIS support identification of usability issues different users encounter when attempting to complete ACT SMARTS activities/tasks?
3. To what extent does the CWIS methodology support identification of which ACT SMARTS activities are most problematic or difficult for users to complete?
4. To what extent does the CWIS methodology inform support further redesign to components of ACT SMARTS to maximize usability and fit in schools?

### Methodology and Case Study Application

#### Adoption of Curricular supports Toolkit: Systematic Measurement of Appropriateness and Readiness for Translation in Schools (ACT SMARTS) Case Study Application

**Figure 1:**
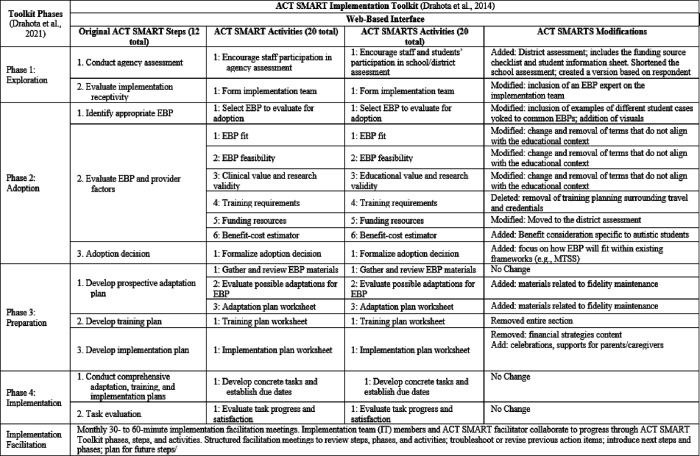
ACT SMARTS Modification Blueprint –Redesign areas to ACT SMART phases and activities for use in schools

As mentioned, we applied the CWIS approach to create the redesigned ACT SMARTS for use in middle and high schools. ACT SMARTS is a packaged, multifaceted selection-quality implementation toolkit strategically targeting the identification and selection of appropriate autism evidence-based practices (EBPs) selection and decision-making (18). Originally developed in collaboration with a community-academic partnership for use in community-based autism-focused organizations, preliminary data support its feasibility, acceptability, utility, and effectiveness in such settings (19,20). However, redesign was needed to enhance its fit and utility in public schools given the unique implementation context of schools and importance of context-specific fit (21,22). The goal of this project was to use the novel CWIS methodology as part of the iterative, systematic redesign of this original toolkit to create ACT SMARTS. Two frameworks guided this broader project. First, the Exploration, Preparation, Implementation, and Sustainment (EPIS; 18,19) framework is the overarching framework given its application during the development of and integration in the original toolkit. Additionally, we apply the Discover, Design, Build, and Test (DDBT; 20) framework to guide the iterative redesign process. The initial redesign methods, procedures, and full description of redesigned ACT SMARTS prototype are described in Locke et al. 2024 (18). In brief, we engaged community members (i.e., educators, administrators, autistic individuals, caregivers of autistic youth) in mixed-method data collection sessions consisting of focus groups that also consisted of survey collection to identify perceptions and necessary areas for redesign of the original toolkit to optimize its feasibility and utility in middle and high schools. Results informed iterative redesign and development of a modification blueprint of ACT SMARTS (See Figure 1).

ACT SMARTS includes a school-based implementation team comprised of principals or other leaders and educators involved in EBP selection, phase-specific activities, and facilitation. It has four implementation phases with corresponding steps and activities: 1) Exploration: a district, school, and autistic student needs assessment evaluating organizational determinants (e.g., implementation climate, organizational readiness) and student and professional needs. 2) Adoption:activities to support the identification and evaluation (e.g., feasibility, fit) of relevant EBPs that match needs and determinants identified in Phase 1. This phase culminates with a formal EBP adoption decision made by the school-based implementation team. 3) Preparation: activities and tools to guide planning for EBP adaptation, staff training, and EBP implementation. 4) Implementation:EBP implementation, guided by products developed during the prior preparation phase such as the adaptation and implementation plan, and an evaluation of implementation efforts. The ACT SMARTS implementation facilitator systematically supports the school-based implementation team as they carry out the ACT SMARTS phase-specific activities and tasks. The current work represents the next step in this multiphase project where we subjected the redesigned ACT SMARTS prototype to further user testing to evaluate its usability to inform further refinements prior to conducting a feasibility pilot test in middle and high schools

#### Step 1: Determine preconditions of the implementation strategy

Consistent with the first step of CWIS, four members, including study PIs with expertise in EBP implementation in schools, the original developer of the implementation toolkit and a graduate student with expertise in the implementation toolkit, collectively determined preconditions at both the individual, school, and district levels. Individual or educator level preconditions included those involved in the selection and/or delivery of autism EBPs in their school and access to resources as EBP supports (e.g., training or education, funding). School preconditions similarly included leaders involved in the selection of autism EBPs in their schools and access to resources as EBP supports. District preconditions include administrators involved in the selection of autism EBPs and identifying or allocating resources as EBP supports.

#### Step 2: Hierarchical Task Analysis

We then conducted a hierarchical task analysis by identifying all tasks and subtasks that comprised ACT SMARTS’ four phases (i.e., Exploration, Adoption, Preparation, Implementation). Four members of the study team, including the PIs, the original developer of the toolkit, and an additional doctoral level graduate student with expertise with the original implementation toolkit participated in this step. We identified corresponding user groups, with some tasks unique to one end user group (e.g., the funding checklist only relevant to principals or other leaders) and others relevant tasks across user groups. We then articulated, reviewed, and revised specified tasks to generate a complete list of tasks associated with completion of the ACT SMARTS process.

#### Step 3: Task Prioritization

As part of task prioritization, we prioritized the specific ACT SMARTS tasks to include in subsequent user testing sessions, including reviewing and rating the potential tasks on the anticipated likelihood of an issue or committing an error and importance of completing a task correctly. After combining ratings, the highest rated tasks were selected and prioritized for user testing based on completion importance followed by likelihood of issue/error. We selected 9 tasks for conversion to scenarios and subtasks, with 6 subtasks chosen for district administrators, 9 subtasks for school principals, and 8 subtasks for educators.

#### Task 4: Convert top tasks to testing scenarios

We converted selected tasks to scenarios and subtasks (Step 4) for each user type (i.e., district administrator, school administrator, educator). Consistent with CWIS, we crafted overarching scenarios from task themes to provide background context on settings and activities to aid users to cognitively illustrate how, when, where, and with whom its subtasks were to occur during user testing. Subtasks within scenarios were created around related prioritized tasks. We made revisions to tasks through an iterative process to ensure that scenarios will be independent of one another and that subtasks will be discrete, achieved through the expansion, combination, and operationalization of prioritized tasks. See Table 1 for full listing of scenarios and subtasks.

#### Task 5: User Testing

We then conducted group testing (Step 5) across three virtual sessions (range = 3–6 participants) where participants assumed the ACT SMARTS district administrator (n = 3), school administrator (n=6), or educator (n = 6) user types. Participants were assigned to a two-hour session with an associated user type based on their availability. One week prior to their session, participants received a digital copy of ACT SMARTS materials tailored to each user type, user type’s scenarios and subtasks to review, and a disclosure form to complete. Each user testing session had facilitators, a notetaker, and a technology assistant. Project PIs assumed the role of facilitators and notetakers and the study coordinator assumed the role of technology assistant. Following a standard script, the facilitator began each session with an orientation to the project including an overview of ACT SMARTS, the user testing session, and participation expectations. The cognitive walkthrough process consists of the presentation of a scenario and its subtasks, quantitative ratings and qualitative rationales from participants, and open-ended discussion by participants. For each subtask, a written description was visually presented on a PowerPoint along with an image that depicted the subtasks’ intent. We also provided a unique link to access the previously shared digital materials for each subtask so participants could reference during user testing sessions. We asked all participants to share their quantitative likelihood of success ratings and associated rationales for each subtask. After each participant provided their ratings and justifications, the facilitator encouraged open discussion about potential barriers and facilitators for the subtask. Following the completion of all scenarios and subtasks, the facilitator presented three open-ended questions to the group designed to capture additional comments about potential usability issues. These included: What was your overall impression of ACT SMARTS? How does this compare to other implementation strategies or supports to promote the adoption of new programs/practices that you’ve been exposed to? Is there anything else that you would like to share about your experience today or with ACT SMARTS more generally? Lastly, participants completed the 10-item Implementation Strategy Usability scale (26). Participants received a $200 gift card for their participation.

#### Task 6: Usability issue identification, prioritization and classification

Results from user testing directly informed our identification, classification, and prioritization of usability issues as outlined in Step 6 of the CWIS process. Employing the guidance specified by the University of Washington ALACRITY Center (27) applied in the original description of CWIS, two members of the research team (first and second authors) specified the usability issues resulting from our group testing. This included creating brief usability statements and rating the severity, scope, complexity, and consequences. Identified usability issues directly informed subsequent modifications and further redesign of ACT SMARTS to enhance its usability.

### User Testing Recruitment

The Institutional Review Boards of San Diego State University and the University of Washington approved the study. We created a stratified recruitment plan using a free, online platform (https://www.thegeneralizer.org/; 21), selecting specific school characteristics (e.g., School Size, % Free & Reduced Lunch, % Female, Urbanicity, % White, % Black, % Hispanic, % Two or More Races School Count, % English Language Learner, % English Only, % Other Than English) to ensure a representative sample. We categorized schools into five strata groups within California and Washington, where we conducted this study, closely mirroring the composition of the target population. We compiled a ranked list of schools based on five strata categories, including relevant corresponding school names, districts, and other demographic information. We contacted identified schools and invited them to participate, sending 4,935 recruitment emails including all study materials (e.g., flyers, IRB approvals, screener materials) to principals and educators from the strata lists; this resulted in 76 interest forms across all participants (response rate of 1.54%).

**Figure 2:**
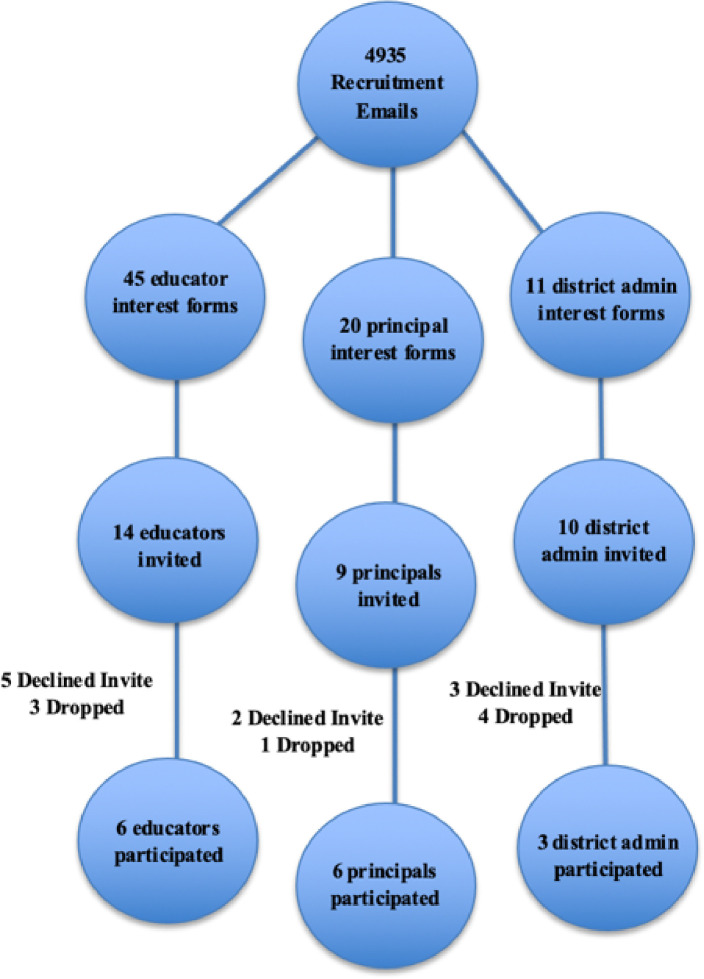
Recruitment and Enrollment Flow Chart *Note*. “Declined Invite” are those who were invited but did not respond to the invitation email and did not consent to participate in the group. “Dropped” are those who accepted the invitation to participate in the focus group, completed the consent, but were unable to attend the group.

We considered various factors in participant selection, with preference given to individuals whose availability overlapped with other participants. Furthermore, participant selection considered educator role, school strata, gender, race, and ethnicity to ensure the creation of representative and diverse groups. Of the 76 interest forms received, 45 were general and special education teachers, 20 principals, and 11 district administrators. We invited 14 general and special education teachers to participate and five of those participants either did not respond or were unavailable on the day of the CWIS session. We invited nine principals to participate and two declined due to not being available on the date of the CWIS session. We invited 10 district administrators to participate and three declined the invitation due to scheduling conflicts or did not respond. Before participation, the research team provided a full description to all participants of study procedures and activities included in study participation. For more details on recruitment and enrollment, see Figure 2.

### Participants

Four district administrators, three educators, and one principal were unavailable for their scheduled and confirmed CWIS session due to emergencies that came up the day of the session; all other invited participants attended their scheduled CWIS session. Participants (n=15) included school district administrators (n=3), middle and high school principals (n=6), general (n=4) and special education (n=2) teachers. Participants represented 13 school districts and 12 middle and high schools across California and Washington. Participants were primarily female (86.7%), White (73.3%) and Non-Hispanic, Latino or of Spanish Origin (73.3%), the average number of years working in a school was 19.5 years, and the average number of years working with autistic individuals was 15.9 years. The majority of educators in our study identified as White, although, this reflects the broader demographics of educators in the states where the study occurred. Educators are reported to be 93% and 63% White, respectively in these states, according to the Institute of Education Sciences (29) and the California Department of Education (30). The diverse range of participant experiences (e.g., geographic area, age, years of experience) offered a valuable opportunity to collect insights from distinct perspectives (31). For more detailed participant demographics, refer to Table 2.

### Measures

#### Implementation Strategy Usability Scale (ISUS; 29)

User testing participants completed the ISUS during user testing (CWIS Step 5). The ISUS is an adapted version of the well-established System Usability Scale (32,33), a 10-item measure of the usability of digital technologies. Odd items (1, 3, 5, 7, 9) are totaled and then five is subtracted. Even numbers (2, 4, 6, 8, 10) are totaled and then subtracted from 25 to account for reverse scoring. The two numbers are then added together and multiplied by 2.5 to obtain a final usability score. Scores range from 0–100 with <50 indicating unacceptable usability and >70 acceptable. Used in more than 500 studies, the SUS is the best-researched usability measure (33,34).

#### Anticipated Likelihood of Success

User testing participants completed quantitative items assessing their anticipated likelihood of success for each subtask presented during user testing (CWIS Step 5). These included the following items: (1) knowing what to do (“discovering that the correct action is an option”), (2) doing it (“performing the correct action or response”), and (3) knowing they did it successfully (“receiving sufficient feedback to understand that they have performed the correct action”). Participants rated items on a scale of “1” (No, a very small chance of success) to “4” (Yes, a very good chance of success) (10).

### Analysis.

Analyses focused on the mixed-methods collected during user testing and consisted of a two-step process. First, we calculated descriptive analyses of all likelihood of success ratings, flagging ratings with ratings below a 3 (probable chance of success). We also calculated ISUS following the scoring instructions from (10). Next and consistent with the rapid qualitative assessment process (35), we identified emergent themes from our qualitative data from each participant. Rationales were transferred to templates, organized by identified tasks and subtasks, with those tasks or subtasks included across user types grouped together, and linked with quantitative ratings of success. Together, quantitative and qualitative ratings informed our identification of usability issues regarding the ACT SMARTS (Step 6). Consistent with the CWIS methodology, we developed a description of the issue and classified the corresponding severity, scope, level of complexity, and problem type to identify and sort usability issues. Qualitative themes and usability issues were reviewed and validated by a team member not involved in the coding. The resulting usability issues provided insight into the feasibility and contextual appropriateness of the ACT SMARTS prototype for each user type (i.e., district or school administrator, educator, school-based clinician). Lastly, our team collectively developed redesign solutions for the ACT SMARTS based on the usability issue descriptions and evidence provided

### Results

#### ACT SMARTS Anticipated Likelihood of Success.

**Figure 3:**
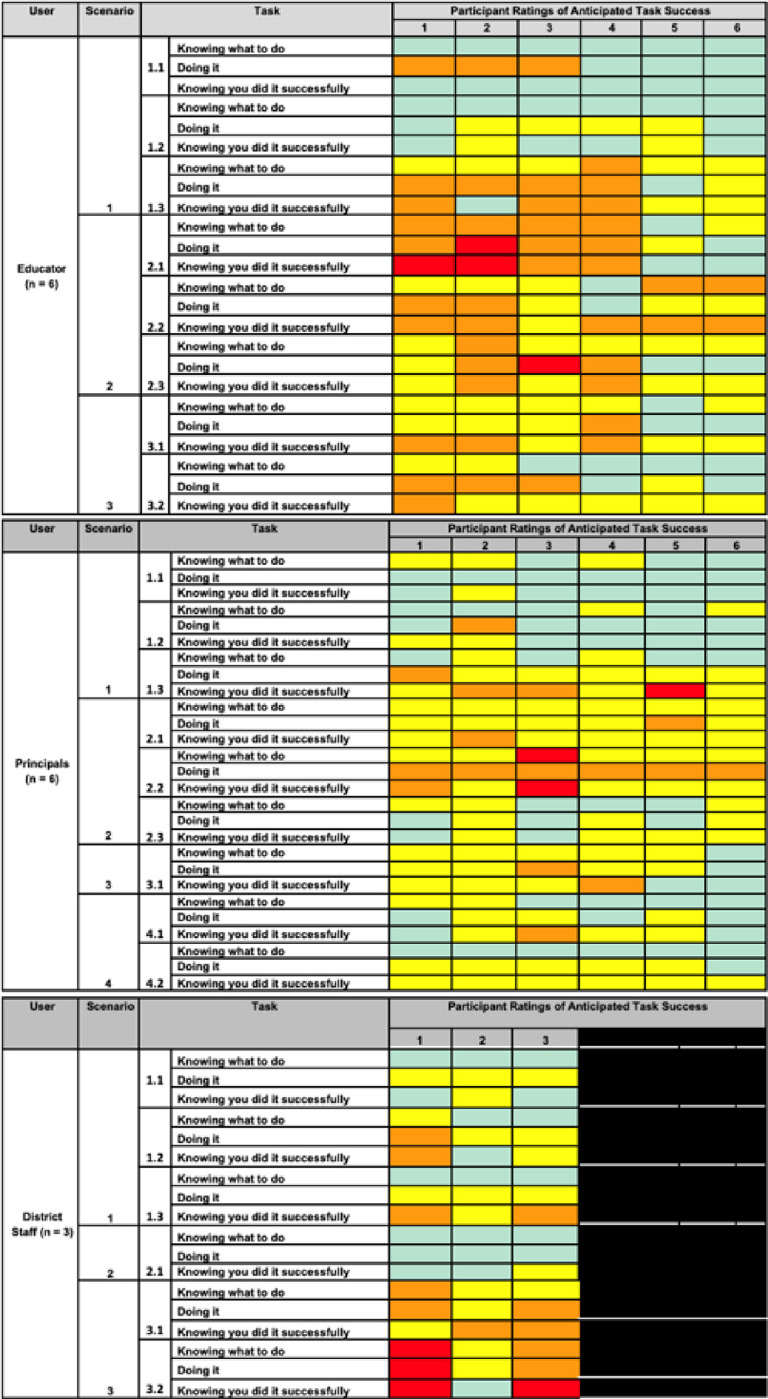
Anticipated Likelihood of Success Rating

Results from the participants’ anticipated likelihood of success ratings for each subtask, as presented during user testing, are illustrated in Figure 3. The ratings are color-coded to visually represent the scale utilized for our likelihood of success ratings, with “1” indicating “No, a very small chance of success” (depicted in red), “2” indicating “No, probably not” (depicted in orange), “3” indicating “Yes, probably” (depicted in yellow), and “4” indicating “Yes, a very good chance of success” (depicted in green). Overall, educators indicated a higher anticipated success of *knowing what to do* (*M* = 3.23, *SD* = 0.72) with regard to completing ACT SMARTS tasks than *doing it* (*M* = 2.83, *SD* = 0.91) and *knowing they did it successfully* (*M* = 2.85, *SD* = 0.87). As seen in Figure 3, there was significant variation in the ratings across subtasks, with high likelihood of completing tasks associated with scenario 1 tasks of ACT SMARTS (e.g., completing a school assessment) and lower perceived likelihood of completing scenario 2 tasks (i.e., implementation plan development). Principals indicated a higher likelihood for anticipated success than educators, with *knowing what to do* (*M* = 3.33, *SD* = 0.63), *doing it* (*M* = 3, *SD* = 0.68), and *knowing they did it successfully* (*M* = 3, *SD* = 0.63) between “Yes, probably” and “Yes, a very good chance of success,” with an overall average across subtasks and factors of 3.11 (*SD* = 0.66). Again, there was similar variation across the ratings with performing the action and receiving sufficient feedback to know they performed the correct action scoring slightly lower than identifying the correct action. Finally and similar to educator participants, district administrators indicated higher anticipated success *knowing what to do* (*M* = 3.39, *SD* = 0.92) to complete ACT SMARTS tasks than *doing it* (*M* =2.83, *SD* =0.79) and *knowing they did it successfully* (*M* = 2.83, *SD* = 1.04); ratings were generally between “Yes, probably” and “Yes,” with an overall average across subtasks and factors of 3.02 (*SD* = 0.94). Again, there was similar variation across the ratings with performing the action and receiving sufficient feedback to know they performed the correct action scoring lower than identifying the correct action. In terms of our Research Question 1, results suggest that the application of CWIS aided in identification of users understanding, with users demonstrate a general, albeit variable, understanding or knowledge of what to do in terms of the ACT SMARTS activities but more limited anticipated success doing the task and knowledge they completed it successfully. Results also pointed to key tasks or activities that are more problematic for users to complete (Research Question 2), including completion of the Phase 1 school assessment, Phase 2 EBP Benefit/Cost estimator, and Phase 3 adaptation and implementation plans.

#### ACT SMARTS Usability.

In terms of Research Question 2, CWIS aided in the supported the identification of ACT SMARTS usability issues. Specifically, ISUS results indicated variable perceptions from all participants regarding the usability of the ACT SMARTS, higher average usability ratings from educators (Mean = 45.83; SD= 13.93; Range = 42.50 – 65.00) and district administrators (Mean = 55.83; SD = 15.07; Range=40.00 – 70.00) than principals (Mean=52.50; *SD* = 9.49; Range = 22.50 – 62.50). According to the standards put forth by Bangor et al. (2008), the average educator user type rating was indicated to be between “poor” (1^st^ quartile) and “ok” (2^nd^ quartile). The average principal and district user type rating was between “ok” (1^st^ quartile) and “good” (2^nd^ quartile). These scores all fall into the “marginal” range. Table 3 ISUS lists the usability ratings for ACT SMARTS.

#### ACT SMARTS Usability Problems and Redesign Solutions.

Pertaining to Research Question 3, results from our CWIS user testing greatly informed the identification of 11 key usability issues of the ACT SMARTS. Many issues were identified across user types (e.g., limited knowledge and experience limiting educator and principal development and use of the implementation plan, limited appropriate staff making principals and district administrators unable to nominate enough staff to complete the school assessment). Across all ACT SMARTS phases and tasks, we identified usability issues involving the following: 1) limited knowledge or experience, or alignment with their professional role; 2) competing professional demands and/or limited compelling rationale for completion; 3) the need to involve others or gather additional information; 4) not having enough relevant staff to nominate for the school or district assessment; 5) volume of information and/or amount of effort required; 6) challenges interpreting results to inform EBP selection and decision making; and 7) challenges identifying EBP benefits prior to its selection. Usability issues ranged low-medium in complexity (“solutions are clear and feasible” to “solutions are somewhat unclear”) and all but one had severity ratings that ranged from “creates significant delay and frustration” to “has a minor effect on usability.” Our usability issue regarding challenges identifying the benefits of an EBP prior to its selection had a higher severity rating of “prevents completion of a task.” Consistent with CWIS, usability issues informed the identification and subsequent execution of redesign solutions to enhance the usability of the ACT SMARTS in schools (Research Question 4). We identified redesign solutions for each usability issue and included: 1) embed ACT SMARTS activities into existing school structures (e.g., meetings, professional development); 2) integrate engagement strategies to promote buy-in and completion of tasks; 3) emphasize completion of ACT SMARTS tasks during facilitation meetings to ensure appropriate knowledge and expertise and efficient completion; 4) simplify task requirements; 5) proactively notify end users of task requirements to ensure gathering of materials and/or expertise prior to completion; and 6) adjust the sequence of ACT SMARTS tasks to ensure successful completion. See Table 4 for full listing of usability issues and corresponding ratings and redesign solutions.

## Discussion

Here we present a case study applying the CWIS (10) to identify key usability issues in service of informing further redesign of ACT SMARTS, a multifaceted selection-quality implementation toolkit, for its use in middle and high schools. Our results confirmed that our CWIS application highlighted users’ understanding of ACT SMARTS activities and tasks (Research Question 1) and specific usability issues completing tasks (Research Question 2). Additionally, CWIS greatly aided in the identification and articulation of key user issues for specific ACT SMARTS activities that were problematic and/or challenging to complete (Research Question 3) as well as corresponding redesign solutions to enhance its potential usability in schools (Research Question 4). Overall, our findings highlight the immense potential to use this methodology to inform the design and development of implementation strategies to enhance their effectiveness in key community settings such as schools.

As mentioned, CWIS demonstrated significant utility in not only the identification but also the articulation of the specific usability issues associated with the ACT SMARTS prototype, particularly those that likely contributed to discontinued use of ACT SMARTS if not addressed. For example, users identified significant challenges completing our EBP benefit/cost estimator prior to the selection of a specific EBP during the ACT SMARTS process. Challenges completing this task prior to the selection of an EBP also emerged in prior work evaluating the original selection-quality implementation toolkit, where fidelity or adherence to and completion of this particular task was lower compared to other specified activities and tasks (19). The identification of this usability issue informed development and execution of a redesign solution adjusting the order or sequencing of this ACT SMARTS activity, so an EBP is selected prior to the completion of the benefit/cost estimator. There is an identified need to intentionally design and tailor implementation strategies to enhance their impact (2), and our results point to CWIS being an effective and valuable method to inform the development and modification of implementation strategies tailored for the specific deployment contexts. The identification of usability issues and corresponding redesign solutions ultimately enhances the utility and potential impact of ACT SMARTS.

Results from our user testing indicated variable but overall marginal usability ratings, consistent with our prior initial redesign work that informed the development of our ACT SMARTS prototype, noting strong appropriateness and acceptability but more moderate feasibility and usability (18). These ratings are, however, lower than those observed in prior similar work applying CWIS (10,16). The lower usability ratings may be due to the multifaceted, multilevel, and comprehensive nature of ACT SMARTS compared to implementation strategies of focus in prior CWIS work that consists of less comprehensive multifaceted implementation strategies with fewer components and/or associated activities. Given the importance of strategic and systematic inclusion of strategies matched to specific contexts and determinants (2), our usability results prompted evaluation of the need for the full spectrum of tasks and activities detailed in ACT SMARTS. Importantly, our current work as well as prior redesign work support the value and need for the comprehensive nature of ACT SMARTS. However, further redesign to enhance its usability is needed. Specifically, our user testing results indicated that participants perceived ACT SMARTS activities or tasks as necessary or helpful for EBP implementation, many are novel and/or outside their expertise or experience in ways that led to challenges in completion. Again, CWIS demonstrated utility in both identifying this as a usability challenge and informing our development of a redesign solution of shifting the completion of these ACT SMARTS tasks to occur within the context of the implementation team and/or facilitation to ensure effective and successful completion.

In terms of our process applying CWIS, several considerations and/or supports were critical to our successfully completing the prescribed, connected steps detailed as part of the CWIS process. Although this is an independent application of CWIS, we received training and consultation from its developers which was key to our application. We received guidance in the comprehensive development of necessary preconditions and hierarchical task analysis. Most notably, we sought additional consultation regarding the development of effective, appropriate tasks and testing scenarios to optimize our user testing and in the identification and classification of usability issues. For usability classification, we particularly struggled with the classification of usability issues and specific problem types as those currently specified did not fully capture or articulate those encountered in our application that ultimately required additional consultation from CWIS developers. Finally, we observed that user testing participants were appropriately engaged in our user testing sessions but initially experienced challenges engaging in the cognitive walkthrough. However, and per recommendations from the CWIS developers, we had participants complete an initial unrelated scenario and associated tasks to orient them to the walkthrough process. Ultimately, this example scenario was essential to ensuring all participants fully understood and appropriately engaged in subsequent walkthroughs. Together, this points to some additional considerations, resources, and areas for further specification, particularly those that alleviate the need for ongoing involvement of CWIS developers in future applications.

The application of CWIS to our ACT SMARTS prototype resulted in identification of several key usability issues and corresponding redesign solutions to enhance its utility and impact when used in middle and high schools. Following completion of this process, we integrated our developed redesign solutions into our ACT SMARTS prototype in preparation for a subsequent pilot feasibility test further examining the implementation and clinical effectiveness of this redesigned toolkit within middle and high schools. This pilot test is currently underway in several schools throughout California and Washington.

### Limitations

As with all studies, this case study has several limitations that should be noted when interpreting the results. Although we employed several best-practice methods for ensuring a representative sample of schools and districts across the United States, recruitment was limited to two Western United States regions. Additionally, we had a relatively modest sample size for our user testing that, while consistent with prior applications of CWIS and the broader human-centered design field, could impact the generalizability of findings to the wider population. While the current study represents one of the first independent applications of the CWIS methodology outside of the original developers, we received training and consultation from the developers as described above which impacted our application and results from this process. Lastly, user testing was only conducted once during the larger iterative redesign of the ACT SMARTS process.

## Conclusions

Implementation strategies are critical to improving the adoption, implementation and scale-up of new innovations and there is a need to systematically and intentionally design and tailor these strategies for their specific deployment context to optimize their effectiveness. As a result, there is a critical need for effective, pragmatic methods to design and tailor implementation strategies. CWIS is a promising method for assessing implementation strategy usability to inform further tailoring and redesigning for deployment. The current work describes the application of the CWIS method to iteratively redesign a multifaceted implementation toolkit for use in middle and high schools. Overall, our results highlight the immense utility of CWIS in service of yielding an enhanced, usable implementation toolkit for use in schools. Additional considerations and recommendations for the continued application of CWIS are also presented.

## Data Availability

Data will be made available upon request to the corresponding author.

## Abbreviations

CWIS: Cognitive Walkthrough for Implementation
HCD: Strategies Human-Centered Design
ACT SMARTS: Adoption of Curricular supports Toolkit: Systematic Measurement of Appropriateness and Readiness for Translation in Schools
EBPs: Evidence-Based Practices
EPIS: Exploration, Preparation, Implementation, and Sustainment
DDBT: Discover, Design, Build, and Test
ISUS: Implementation Strategy Usability Scale
